# Supplementary material for: Mothers’ perspectives of the barriers and facilitators to reducing young children’s screen time during COVID-19: A reddit content analysis
Source: PLoS One. 2024 Mar 27;19(3):e0301089. doi: 10.1371/journal.pone.0301089 (PMC10971781; doi:10.1371/journal.pone.0301089)
Supplement: Attachment — Submitted filename: PONE-D-23-18207.pdf [file pone.0301089.s001.pdf]

PLOS ONE

Mothers' Perspectives of the Barriers and Facilitators to Reducing Young Children's Screen Time During COVID-19: A Reddit Content Analysis

--Manuscript Draft--

**Manuscript Number:**	PONE-D-23-18207
**Article Type:**	Research Article
**Full Title:**	Mothers' Perspectives of the Barriers and Facilitators to Reducing Young Children's Screen Time During COVID-19: A Reddit Content Analysis
**Short Title:**	A Reddit Content Analysis of Mothers' Perspectives on Reducing Young Children's Screen Time During COVID-19
**Corresponding Author:**	Leigh M. Vanderloo, Ph.D.<br>University of Western Ontario<br>London, Ontario CANADA
**Keywords:**	screen time; COVID-19; mothers; barriers; facilitators; young children
**Abstract:**	Screen time for children under five is associated with various health risks. Amidst the COVID-19 pandemic, screen use among young children increased significantly. During COVID-19, mothers were more likely than fathers to be the primary caregivers and disproportionally assumed the responsibility of monitoring their children's screen time. Several studies have examined children's screen use throughout the pandemic; however, few have addressed mothers' experiences on this subject. Therefore, the current study aimed to investigate mothers' perceptions regarding the barriers and facilitators they faced when trying to reduce their child's pandemic screen time, as expressed on Reddit (social media platform for anonymous discussion and information sharing). Two subreddit forums targeted toward mothers, "mommit" and "beyondthebump," with 646,000 and 554,000 users, respectively, were examined. Posts were collected using related search terms and screened for inclusion by three independent researchers. The knowledge creation component of the Knowledge to Action (KTA) framework was used to refine the broad scope of data. Per the knowledge synthesis phase of the framework, emergent themes were devised using inductive thematic content analysis. Posts were coded and divided into relevant themes. A total of 582 individual posts were reviewed from March 14th, 2020, to August 31st, 2022. Qualitative analysis yielded ten preliminary themes; nine barriers and four facilitators were derived from themes and/or subthemes, where applicable. These results showed consistency with previous findings that parents faced new challenges when trying to reduce their child's pandemic screen time. If future interventions utilize the conclusions of this study to address what mothers perceive to be helping or hindering them, mothers may be empowered to successfully limit their child's screen time.
**Order of Authors:**	Leigh M. Vanderloo, Ph.D.
	Leann Blake
	Patricia Tucker
**Additional Information:**	
**Question**	**Response**
**Financial Disclosure**<br><br>Enter a financial disclosure statement that describes the sources of funding for the work included in this submission. Review the submission guidelines for detailed requirements. View published research articles from *PLOS ONE* for specific examples.	The author(s) received no specific funding for this work.

Powered by Editorial Manager® and ProduXion Manager® from Aries Systems Corporation

This statement is required for submission and **will appear in the published article** if the submission is accepted. Please make sure it is accurate.<br><br>**Unfunded studies**<br>Enter: *The author(s) received no specific funding for this work.*<br><br>**Funded studies**<br>Enter a statement with the following details:<br>• Initials of the authors who received each award<br>• Grant numbers awarded to each author<br>• The full name of each funder<br>• URL of each funder website<br>• Did the sponsors or funders play any role in the study design, data collection and analysis, decision to publish, or preparation of the manuscript?<br>• **NO** - Include this sentence at the end of your statement: *The funders had no role in study design, data collection and analysis, decision to publish, or preparation of the manuscript.*<br>• **YES** - Specify the role(s) played.<br><br>* typeset	
**Competing Interests**<br><br>Use the instructions below to enter a competing interest statement for this submission. On behalf of all authors, disclose any competing interests that could be perceived to bias this work—acknowledging all financial support and any other relevant financial or non-financial competing interests.<br><br>This statement is required for submission and **will appear in the published article** if the submission is accepted. Please make sure it is accurate and that any funding sources listed in your Funding Information later in the submission form are also declared in your Financial Disclosure statement.<br><br>View published research articles from	The authors have declared that no competing interests exist.

PLOS ONE for specific examples.<br><br>**NO authors have competing interests**<br><br>Enter: *The authors have declared that no competing interests exist.*<br><br>**Authors with competing interests**<br><br>Enter competing interest details beginning with this statement:<br><br>*I have read the journal's policy and the authors of this manuscript have the following competing interests: [insert competing interests here]*<br><br>* typeset	
**Ethics Statement**<br><br>Enter an ethics statement for this submission. This statement is required if the study involved:<br><br>• Human participants<br>• Human specimens or tissue<br>• Vertebrate animals or cephalopods<br>• Vertebrate embryos or tissues<br>• Field research<br><br>Write "N/A" if the submission does not require an ethics statement.<br><br>General guidance is provided below. Consult the submission guidelines for detailed instructions. **Make sure that all information entered here is included in the Methods section of the manuscript.**	N/A

**Format for specific study types**<br><br>**Human Subject Research (involving human participants and/or tissue)**<br>• Give the name of the institutional review board or ethics committee that approved the study<br>• Include the approval number and/or a statement indicating approval of this research<br>• Indicate the form of consent obtained (written/oral) or the reason that consent was not obtained (e.g. the data were analyzed anonymously)<br><br>**Animal Research (involving vertebrate animals, embryos or tissues)**<br>• Provide the name of the Institutional Animal Care and Use Committee (IACUC) or other relevant ethics board that reviewed the study protocol, and indicate whether they approved this research or granted a formal waiver of ethical approval<br>• Include an approval number if one was obtained<br>• If the study involved *non-human primates*, add *additional details* about animal welfare and steps taken to ameliorate suffering<br>• If anesthesia, euthanasia, or any kind of animal sacrifice is part of the study, include briefly which substances and/or methods were applied<br><br>**Field Research**<br><br>Include the following details if this study involves the collection of plant, animal, or other materials from a natural setting:<br>• Field permit number<br>• Name of the institution or relevant body that granted permission	
**Data Availability**<br><br>Authors are required to make all data underlying the findings described fully available, without restriction, and from the time of publication. PLOS allows rare exceptions to address legal and ethical concerns. See the PLOS Data Policy and FAQ for detailed information.	Yes - all data are fully available without restriction

A Data Availability Statement describing where the data can be found is required at submission. Your answers to this question constitute the Data Availability Statement and **will be published in the article**, if accepted.<br><br>**Important:** Stating 'data available on request from the author' is not sufficient. If your data are only available upon request, select 'No' for the first question and explain your exceptional situation in the text box.<br><br>Do the authors confirm that all data underlying the findings described in their manuscript are fully available without restriction?	
**Describe where the data may be found in full sentences. If you are copying our sample text, replace any instances of XXX with the appropriate details.**<br><br>• If the data are **held or will be held in a public repository**, include URLs, accession numbers or DOIs. If this information will only be available after acceptance, indicate this by ticking the box below. For example: *All XXX files are available from the XXX database (accession number(s) XXX, XXX.)*.<br>• If the data are all contained **within the manuscript and/or Supporting Information files**, enter the following: *All relevant data are within the manuscript and its Supporting Information files.*<br>• If neither of these applies but you are able to provide **details of access elsewhere**, with or without limitations, please do so. For example:<br><br>*Data cannot be shared publicly because of [XXX]. Data are available from the XXX Institutional Data Access / Ethics Committee (contact via XXX) for researchers who meet the criteria for access to confidential data.*<br><br>*The data underlying the results presented in the study are available from (include the name of the third party*	Additional data can be obtained without restrictions by contacting the corresponding author.

and contact information or URL).<br>• This text is appropriate if the data are owned by a third party and authors do not have permission to share the data.<br><br>* typeset	
Additional data availability information:	

Manuscript
Click here to access/download;Manuscript;Reddit Study_Blake_submitted.docx

Mothers' Perspectives of the Barriers and Facilitators to Reducing Young Children's Screen Time During COVID-19: A Reddit Content Analysis

Leann Blake[1], Patricia Tucker[2], & Leigh M. Vanderloo[2,3]

1. School of Health Studies, Western University, London, Canada
2. School of Occupational Therapy, Western University, London, Canada
3. ParticipACTION, Toronto, Canada

Corresponding author: Leigh Vanderloo

Abstract

Screen time for children under five is associated with various health risks. Amidst the COVID-19 pandemic, screen use among young children increased significantly. During COVID-19, mothers were more likely than fathers to be the primary caregivers and disproportionally assumed the responsibility of monitoring their children's screen time. Several studies have examined children's screen use throughout the pandemic; however, few have addressed mothers' experiences on this subject. Therefore, the current study aimed to investigate mothers' perceptions regarding the barriers and facilitators they faced when trying to reduce their child's pandemic screen time, as expressed on Reddit (social media platform for anonymous discussion and information sharing). Two subreddit forums targeted toward mothers, "mommit" and "beyondthebump," with 646,000 and 554,000 users, respectively, were examined. Posts were collected using related search terms and screened for inclusion by three independent researchers. The knowledge creation component of the Knowledge to Action (KTA) framework was used to refine the broad scope of data. Per the knowledge synthesis phase of the framework, emergent themes were devised using inductive thematic content analysis. Posts were coded and divided into relevant themes. A total of 582 individual posts were reviewed from March 14th, 2020, to August 31st, 2022. Qualitative analysis yielded ten preliminary themes; nine barriers and four facilitators were derived from themes and/or subthemes, where applicable. These results showed consistency with previous findings that parents faced new challenges when trying to reduce their child's pandemic screen time. If future interventions utilize the conclusions of this study to address what mothers perceive to be helping or hindering them, mothers may be empowered to successfully limit their child's screen time.

Keywords: screen time, COVID-19, mothers, barriers, facilitators, young children

Mothers’ Perspectives of the Barriers and Facilitators to Reducing Young Children's Screen Time During COVID-19: A Reddit Content Analysis

Excessive screen time during early childhood may adversely impact physical, cognitive, and emotional development [1]. For young children, excess screen use is associated with anti-social behaviours, poorer self-regulation and attention difficulties, cognitive and language delays, and obesity that may persist into adulthood [2-5]. To minimize potential health risks, the Canadian Paediatric Society (CPS) does not recommend screen time for children under 2 years of age, apart from video-chatting with caring adults, and suggests limiting screen time to less than 1 hour per day for 2-to-5-year-olds [1].

Public health measures, such as social distancing and stay-at-home orders were introduced to help reduce the spread of the COVID-19 virus [6]. Consequently, children were expected to learn, socialize, and play remotely at home, resulting in substantial increases in screen time among children under 5 years during this period [7]. Specifically, prior to the COVID-19 pandemic, young children (0-5 years) used screens for approximately 1.5 h/day [8]. This number increased to approximately 2.09 h/day, significantly exceeding the recommended screen time for this age group [8, 9].

Parents of young children have considerable control over the activities in which their children engage [10]. Parents may limit screen use by creating “screen-free” zones, promoting alternatives, and reducing their own screen use to model this behaviour [11]. However, this is not always a manageable task. Pre-pandemic, Jarvis and colleagues [12] found that parents face more challenges when trying to limit their child’s screen time than they do in promoting any other health behaviour. This became increasingly difficult during the pandemic due to the

absence of children's regular structured routines and a dearth in available alternatives to screen time [13].

Social media can be used for information or social support during a crisis and played a crucial role in influencing people's health behaviours during COVID-19 [14, 15]. In response to mothers' increased responsibility of managing their children's pandemic health behaviours, including screen time, many leveraged social media to connect with other mothers for support, to generate activity ideas, and to vent their frustrations [16]. One platform utilized for such actions was Reddit. This free social discussion website allows users to exchange with information and post content on forums designed around specific topics called *subreddits* [17]. These subreddits form communities of users that share common interests or contexts, including motherhood and child rearing [18]. Reddit is valuable for qualitative research for several reasons. First, it is the 18th most popular website in the world, giving rise to large amounts of data [17]. Additionally, posts on Reddit are publicly accessible, and users organize themselves into communities based on specific interests, which allows researchers to easily identify and study eligible participants [17]. Deeper textual analysis is also made possible on Reddit as it lacks character restriction, unlike other social media platforms such as Twitter, which has a 280-character limit that restricts the amount of information users can share with their followers [24]. In addition, Reddit is a text-based platform, in contrast to other popular social media platforms like Instagram, which are photo-based, and focus primarily on users sharing images taken in their daily life with their followers or the public, limiting amount of written, expressive content that may be used for analysis [25]. Moreover, Reddit's anonymity allows users to share their feelings openly; this reduces self-report bias and offers higher rates of self-disclosure [17]. Previous research on mental health has indicated that Reddit is a valuable source of data for qualitative analysis due to

the willingness of anonymous users to express their experiences despite stigma or apprehension that may arise when disclosing their honest thoughts and feelings to mental health professionals and/or friends [26]. Since mothers of young children may experience guilt or shame when sharing the challenges they face as a parent, Reddit offers a safe outlet to be open about their experiences without fear of judgment from others [27, 28].

Numerous quantitative studies have explored children's screen use throughout the pandemic; however, many lack insight regarding how to support caregivers to mitigate this challenge [7, 19, 20] In addition, there is a lack of qualitative research that has examined mothers' impressions of their efforts to limit their young children's screen use during COVID-19. Eyler and colleagues [21] explored parents' perceptions regarding their children's physical activity and screen time during the pandemic. However, this study was not specific to mothers' perspectives (parent who is typically reported as being primarily responsible for childcare [22]) and centred on the screen behaviours of children between 5-12 years [21]. Without understanding what mothers perceive to aid or impede them in reducing their child's screen time, future interventions may fail to address critical factors voiced by caregivers who spend the most time with the child. In addition, resources or supports adopted to assist parents based on information specific to 5-to-12-year-olds may not be effective for younger children. While school-aged children increasingly engage in activities independently, the behaviour of those under 5 years is highly dependent on their caregivers as they require constant entertainment and supervision [23]. As such, by leveraging posts on Reddit, this study aimed to qualitatively understand mothers' perspectives on the barriers and facilitators to reducing their young children's (0-5 years) screen time during the COVID-19 pandemic.

Methods

Approval from the university's ethics review board was not required as all published data were in the public domain.

Posts on Reddit related to screen use were queried and extracted from two subreddit groups for mothers, "Mommit" and "Beyondthebump," with 688,000 and 570,000 users, respectively. Posts were collected using the search terms "screen time," "tablet," "phone," "television," and "TV." Approval from the university's ethics review board was not required as all published data were in the public domain.

Inclusion Criteria

An exploratory, data-driven, content analysis was employed, meaning there were no preconceived hypotheses regarding what would be discussed or shared in the posts [29]. Instead, posts were chosen based on the following inclusion criteria: 1. written by someone who identifies as a mother, 2. discussing their young child's (0-5 years) screen use, and 3. posted between March 14th, 2020, and August 31st, 2022. These dates represent the time between the initial public-school closures in Ontario and the formal commitment by the Ontario government to keep students in school beginning September 2022, including the reintroduction of extracurricular activities [30, 31]. In an attempt to be all-encompassing, Ontario was chosen as the model for the pandemic timeframe, given that this Canadian province had the most prolonged pandemic health restrictions in North America [32]. Three independent researchers screened each post for inclusion to ensure consensus. Reddit posts that were gathered during data collection that did not meet the three inclusion criteria described above were excluded and not utilized for subsequent analysis. Demographic information was not reported for participants, as both subreddits are anonymous forums.

Thematic Analysis

Following the collection of Reddit posts and subsequent screening for inclusion, inductive thematic content analysis, as described by Braun and Clarke [33], was employed to code posts and derive emergent themes. Inductive thematic analysis involved reading and re-reading each post, systematically identifying and naming the aspect related to screen use, searching for patterns, and organizing the data into themes and subthemes [34]. One researcher individually coded each post and devised emergent themes, reviewing and revising them to ensure intra-coder reliability. Two independent researchers reviewed themes, and slight changes were made to improve the conciseness and clarity of themes.

Themes were presented with representative quotations and themes and subthemes were classified into barriers and facilitators. Barriers were defined as factors that inhibited mothers' ability to limit their children's screen use, and facilitators were factors that supported or empowered mothers in this pursuit. A mind map was created to visually present barriers in a user-friendly manner.

Results

The initial search yielded 582 posts uploaded from March 14th to August 31st, 2022. Of these posts, 157 were excluded as they did not meet the inclusion criteria, leaving 425 unique posts to be analyzed. Following analysis, five themes were clear regarding mothers' perspectives of screen time: 1. mothers' other obligations creating an increase in child's screen use; 2. request for advice/tips on how to reduce child's screen time or looking to receive or give; 3. offering child more screen time to allow themselves to rest or recover, 4. compromising with child and allowing increased screen use, and 5. Mother's screen use creating background or secondary screen exposure for the child. Within these five themes, 17 subthemes (three in theme one, seven

in theme two, three in theme three, two in theme four, two in theme five) were identified which are presented in Table 1.

Theme One

In the first theme, mothers' employment, school, or in-home obligations constrained their ability to occupy their children. Mothers felt overwhelmed and wanted to perform well in work or school and complete house chores (e.g., preparing meals and cleaning), leading them to rely on screen use to keep their children entertained during their competing demands. Or, as some mothers expressed, they used screens as "babysitters" for the child while they completed other tasks.

Table 1

Emerging Themes and Subthemes From Reddit Posts Made by Self-Identifying Mothers Regarding their Young Child's Screen Time.

Theme	Theme Description	Subtheme	Supporting Quotes
**Theme One:** **Mothers' other obligations creating an increase in child's screen use.**	Mothers' employment, school, or in-home obligations constrained their ability to occupy their children. Mothers felt overwhelmed and wanted to perform well in work or school and complete house chores (e.g., preparing meals and cleaning), leading them to rely on screen use to keep their children entertained during their competing demands. Or, as some mothers expressed, they used screens as "babysitters" for the child while they completed other tasks.	1.1 Mother work obligations (e.g., working from home or frontline worker). (n = 11)	"Initially working with my baby was just survival mode. I did whatever kept him happy and calm enough to do work. Unfortunately, the answer is mostly watching Sesame Street for a couple hours a day while he plays in his jumper or activity seat or hangs out on my lap."
			"I am so grateful for The Good Dinosaur on Disney+ [on-screen TV show], using TGD as a babysitter has provided [me with] time for meal making, online grocery shopping, WFH [work from home], and numerous other activities."
		1.2 Mother doing online school. (n = 4)	"We try to keep him away from screens completely, but I'm nervous that with school we might fall into allowing it more easily."

			"Before all of this [school], I was really strict on-screen time…I've tried for the past few weeks to stick to that and just do all of my homework at night but it's killing me staying up till midnight and then waking up at 6 with my little girl."
		1.3 To occupy the child while Mom attends to responsibilities in the home (e.g., cooking, cleaning). (n = 18)	"I try to limit screen time, so I save that for when I'm cooking and cleaning."
			"I will wake my daughter up and have her come to the living room to watch tv while I get the boys breakfast."
**<u>Theme Two</u>: Asking for advice/tips from other mothers on how to reduce screen time or looking to vent, share insight, receive, or give support from community of mothers.**	Mothers utilized the Reddit platform to obtain informational support (e.g., advice/tips on reducing their child's screen time) and emotional or peer support (e.g., encouraging mothers to be kinder to themselves, providing relief that other mothers were facing similar screen related challenges). Mothers broadly understood the importance of limiting screen time for their	2.1 Advice/tips on how to reduce screen time from other mothers (e.g., strategy, rules, routines, technical advice). (n = 49)	"What measures can I put in place that limits screen time to a reasonable level and reduced incessant asking for screens?"
			"Is there an app that makes it possible for me to set screen time [limits] for phones and tablets to kids using just my own phone (of course apps will on their devices will have to be pre-installed)?"

	child's health. However, several mothers wanted more information regarding the type of screens associated with the most significant health risks for their child or whether certain content or purposes of screen time “counted” towards their daily limit (e.g., playing educational games, video chatting with grandparents). Many mothers requested guidance to change their child’s screen behaviour or to obtain reassurance from others who may be experiencing similar challenges with their children. Finally, mothers expressed feeling pressured to reduce their child’s screen time because of the abundance of messaging on social media or from their peers or family members regarding the health risks associated with screen time for children. Such pressure perceived by mothers was often accompanied by feelings of guilt for the mother when they did allow screen time for their child.	2.2 Looking for or sharing options/alternatives to screen time (non-screen alternatives, may be technical). (n = 21)	"Hey hey! If you have kiddos around and need something for them to do that’s not screen time or sleeping. Check out Stoopkid Stories Podcast!"
			“My husband usually fills this time with tv, movies, video games, or phone videos and I’m trying to find a better alternative that we could all enjoy and wouldn’t involve 2 hrs of screen time. Although I am okay with occasional movie watching before bed, I do not want it to be an everyday part of our routine.”
		2.3 Travel concerns regarding screen time. (n = 31)	"My girl is generally pretty happy in the car, but she definitely starts getting cranky around 40 minutes. We can time it to coincide with her nap, but that will only buy us 1-2 hours if we’re lucky. Is it time to invest in an iPad to strap to the headrest? I’m not above screen time."
			“I’m going to be travelling with my 17m old next month and need tips on how to survive the 12h40m plane journey! I’ve researched toys, screen time,

			stickers, fav food etc. but how do I make sure she sits during take-off and landing?"
		2.4 Normalization of screen time, encouraging mothers to be avoid being hard on themselves for their child's screen time. (n = 9)	"Screen time is a very divided topic. There's a lot of shaming around it too. Obviously if your child has become unhealthily addicted, something needs to be done. Never feel bad if you need to distract your kid with a screen, or if they are allowed to have screen time."
			"It's about balance for us. They have some screen time, but also always get outside time to explore and play in the dirt."
		2.5 Inquiring if a behaviour is bad for their child, asking for other mother's opinions on their child's screen time, or inquiring about nuances of risk for screen time (e.g., risk of secondary/background TV, asking about risk associated with certain amounts of screen time). (n = 46)	"Just out of curiosity, what do you all consider as "screen time" when discussing what should be limited for young babies? Do you consider anything that involves any kind of screen (tv, movie, phone, computer etc.) or is it specific to content?"
			"It's [TV] one of the things my 10-week-old loves to stare at, but

			is it any different than staring at windows and interesting lamps?"
		2.6 Concerns about high levels of screen use with another caregiver. (n = 13)	"My husband has a short temper and instead of playing with our son or taking him outside, he gives him screen time half the time so he can play video games."
			"Which brings me to my next grievance - when they [grandparents] do 'babysit' they park him in front of screens."
		2.7 Venting about pressure or guilt felt regarding their child's screen use (e.g., feeling bad for being unsuccessful in their pursuit to limit screen time). (n = 11)	"The guilt just eats me alive. I scroll through social media and see so many moms who look like they are doing so much better than me, and I read so many comments that detail how horrible television is to little kids, especially under the age of two, and how you should never ever let them watch anything or play with your phone or interact with screens at all."
			"I feel like a horrible mother. I feel guilty because I stick my kids in front of a screen for hours on end."

**<u>Theme Three:</u> Mothers allowing child more screen time to have a break or recover from illness.**	Mothers offered their child more screen time to allow themselves to rest or recover. When mothers felt tired from entertaining multiple children, they used screen devices as a tool to occupy their children of different ages (e.g., infants and toddlers). Mothers also used their child's screen time as an opportunity for themselves to take a break or engage in an activity for their own mental or physical health benefit (e.g., psychotherapy, making themselves a meal, exercising).	3.1 Pregnancy with another child or has multiple children. (n = 12)	"We do screen time at my house. I am 7 months pregnant with our second baby, so my toddler gets to watch a movie almost every day."
			"Since I'm trying to break our TV habit, any suggestions for keeping toddler entertained and safe while I put my daughter down, 3 times a day? He'll be 2 in a few weeks, so his attention span is still pretty short."
		3.2 Mother sick or injured (e.g., concussion, COVID-19, flu). (n = 23)	"Don't generally like too much screen time for my 13-month-old daughter... but I am so sick with tonsillitis, and I just don't have the energy to do anything else but lay sweating on the sofa and pat her head while she watches moana."
			"I have been very against allowing any screen time for our baby. She is 4 months old. I am on day three of Covid and I just can't. I gave in and let her watch cartoons so I could just have some rest."

		3.3 Mental health (e.g., exhaustion, depression, anxiety). (n = 40)	"I've depended on the screen [for child] a lot while dealing with PPD [PostPartum Depression]."
			"A couple days ago I had therapy and didn't want her interrupting so for the first time in her life I let a screen be her babysitter."
			"She's [child] watching the screen when I do my daily workout."
**<u>Theme Four:</u> Mothers compromising with child and allowing increased screen use.**	Mothers compromised with their children and allowed increased screen time. Posts included in this theme indicated that mothers were conscious of the screen time their children obtained and often tried to limit screen exposure. However, many mothers shared that they loosened screen time rules for their children during the pandemic. Since public health restrictions resulted in the cancellation of their child's usual	4.1 Pandemic changes in activities or routine resulting in more screen time. (n = 20)	"Since we can't go anywhere, I've been letting her watch as much TV as she wants. I felt guilty about it, but I also didn't have much energy to entertain her all day."
			"You better believe I let my almost 3-year-old have screen time! It's a pandemic, we can't go anywhere, of course I do."

	stimulating or fun activities, mothers offered their child screen time to entertain them or because it was an activity the child could engage in independently. Several mothers expressed that they let their child use screens to incentivize the child to engage in behaviours perceived as non-negotiable (e.g., eating, bathing, trimming nails) or to calm the child when they were restless, fussy, or throwing a tantrum.	4.2 Screen use to soothe/calm child or to encourage child to engage in necessary behaviours (e.g., eating, bathing, cutting nails). (n = 42)	"Turning tv back on the minute we leave the room so she can watch cartoons (we limit screen time to 10 minutes of sesame street only if she's super fussy)."
			"My 18-month-old is tantruming more and more, no surprise, but every once in a while, it takes a turn and he then feels impossible to calm down…Literally the only thing that works seems to be tv, which I do not want to become a thing."
**<u>Theme Five:</u> Background or secondary screen exposure for child.**	Mothers' use of screen devices (e.g., TV, phone) created background or secondary screen exposure for their child. Mothers explained their use of screens for background noise and many sought information regarding the risks of secondary screen usage in children (i.e., wondering if secondary screen exposure was of concern to the child's health).	5.1 Mother watching screen device while holding child or while child played in the same room to occupy mother. (n = 27)	"I will never be on leave again just me and the baby. Soaking in the snuggles and contact naps. Binge watching TV all day while we cycle through feedings, tummy time, and naps."
			"I like to keep the tv on in the background throughout the day. My son is 1 and he doesn't watch it, but I've read that it can impact language. Anyone here played tv all the time? How did your kiddos turn out?"
		5.2 TV or other screen exposure while child sleeps or feeds. (n = 23)	"It was about 12:30 in the morning and me and my husband were sitting on the couch

			watching TV while I breast-feed the baby."
			“We pretty much watch TV all night while we feed him [newborn] and while he sleeps in the bassinet next to us on the couch.”

Note. The number of posts reflected in this table (n = 400) does not equal the total number of posts that met the inclusion criteria and were coded (n = 425). Twenty-five posts did not fit into the five themes above, and insufficient patterns were identified to warrant creation of new themes.

Theme Two

Second, mothers utilized the Reddit platform to obtain informational support (e.g., advice/tips on reducing their child's screen time) and emotional or peer support (e.g., encouraging mothers to be kinder to themselves, providing relief that other mothers were facing similar screen related challenges). Within this theme, seven subthemes were identified, which include the following: seeking tips on how to reduce screen time from other mothers (e.g., strategy, rules, routines, technical advice); inquiring about alternatives to screen time (e.g., podcasts, games); seeking advice to occupy their child during travel without relying on screen use; normalization of screen time amongst mothers, encouraging others to avoid being hard on themselves for their child's screen time; inquiring if a behaviour is bad for their child or about nuances of risk for screen time (e.g., risk of communication applications like Facetime); expressing concern about child's screen time obtained with another caregiver; and venting about pressure or guilt felt regarding their child's screen use (e.g., feeling bad for being unsuccessful in limiting screen time). Mothers broadly understood the importance of limiting screen time for their child's health. For instance, many referenced how screen use could cause damage to their children's eyesight or that it is associated with lack of attention or learning difficulties. However, several mothers wanted more information regarding the type of screens associated with the most significant health risks for their child or whether certain content or purposes of screen time "counted" towards their daily limit (e.g., playing educational games, video chatting with grandparents). In addition, many mothers requested guidance to change their child's screen behaviour or to obtain reassurance from others who may be experiencing similar challenges with their children. Finally, mothers expressed feeling pressured to reduce their child's screen time because of the abundance of messaging on social media or from their peers or family members

regarding the health risks associated with screen time for children. Such pressure perceived by mothers was often accompanied by feelings of guilt for the mother when they did allow screen time for their child.

Theme Three

Third, mothers offered their child more screen time to allow themselves to rest or recover. Three subthemes included the mother being pregnant with another child or being overwhelmed with multiple children; sick or injured (e.g., COVID-19, concussion, tonsilitis); or the mother offering child screen time to take a break for their mental health (e.g., exhaustion, depression, anxiety) or to engage in self-care activities (e.g., use screen as babysitter). When mothers felt tired from entertaining multiple children, they used screen devices as a tool to occupy their children of different ages (e.g., infants and toddlers). Mothers also used their child's screen time as an opportunity for themselves to take a break or engage in an activity for their own mental or physical health benefit (e.g., psychotherapy, making themselves a meal, exercising).

Theme Four

In the fourth theme, mothers compromised with their children and allowed increased screen time. The two subthemes in this category included pandemic changes to routine (i.e., limited alternative activities due to stay-at-home orders and social distancing measures) and the need to soothe or calm the child or encourage the child to engage in necessary behaviours (e.g., eating, hygienic practices). Posts included in this theme indicated that mothers were conscious of the screen time their children obtained and often tried to limit screen exposure. However, many mothers shared that they loosened screen time rules for their children during the pandemic. Since public health restrictions resulted in the cancellation of their child's usual stimulating or fun

activities, mothers offered their child screen time to entertain them or because it was an activity the child could engage in independently. In addition, several mothers expressed that they let their child use screens to incentivize the child to engage in behaviours perceived as non-negotiable (e.g., eating, bathing, trimming nails) or to calm the child when they were restless, fussy, or throwing a tantrum.

Theme Five

Finally, the fifth theme highlights that mothers' use of screen devices (e.g., TV, phone) created background or secondary screen exposure for their child. Two subthemes in this category included mothers' use of screens to occupy themselves while holding their child or while the child played in the same room and watching screens while their child slept or fed. Mothers explained their use of screens for background noise and many sought information regarding the risks of secondary screen usage in children (i.e., wondering if secondary screen exposure was of concern to the child's health).

Mothers' Perspectives on Barriers and Facilitators to Screen Time

From the themes identified in the reddit post analysis, six were labelled as barriers and two facilitators to screen use in young children. The six barriers that mothers identified to challenged their ability to limit their young child's screen time were: mothers' work, school, or homecare obligations; adoption of screens to occupy the child during travel (e.g., airplane, road trip); high use of screen time with other caregivers; mothers offering child more screen time to allow themselves to rest or recover (e.g., self-care practices to reduce exhaustion, mother unable to entertain child while sick with COVID-19); pandemic changes to routine; and using screens to calm or soothe the child or to encourage the child to engage in necessary behaviours (e.g., eating, bathing). A visualization of the barriers is presented in Figure 1.

--- INSERT FIGURE 1 ---

In contrast to the barriers listed above, mothers also identified factors that supported or empowered them to reduce their young child's screen time. The two facilitators included receiving advice on how to limit their child's screen use (e.g., technical advice, sharing rules or routines) and sharing ideas for non-screen alternatives (e.g., audiobooks, podcasts, fun games). Mothers were able to learn what strategies or activities were found to be successful in other households. For example, many received technical advice on how to create a time restraint on their child's device to ensure they were not exceeding their daily screen limit.

Discussion

Amidst the COVID-19 pandemic, public health measures localized children to their homes, and resultingly, led to a surge in screen use reported by this young population [9, 35]. Qualitative content analysis of anonymous Reddit posts made by self-identified mothers provided insight into the factors that aided or hindered them in reducing their child's screen time. Results indicate that mothers found it challenging to balance school, work, or household responsibilities and rest when exhausted or sick without relying on their child's use of screens. As a result of adjustments in their child's routine due to COVID-19 public health measures or, because their child was restless or fussy, mothers also expressed the need to compromise and allow their child more screen time. Moreover, they sought opinions or ideas from other caregivers regarding alternatives for screen time or utilized the platform as a space to vent and share their experiences.

Six barriers mothers faced when trying to limit their child's screen time were identified from the five themes and 17 subthemes that emerged from this study. Additional themes and subthemes may have also contributed to increased child screen use, despite not being identified

as distinct barriers. For example, posts demonstrated the normalization of screen time among mothers; mothers received reassurance from others that screen time was normal for children and that they should not feel bad about allowing it. While such reassurance may not have caused mothers to increase their child's screen time, it is possible that this influence resulted in mothers no longer feeling like they needed to actively reduce it. Moreover, mothers shared that they often watched TV with their child in the same room or used their phone while their child was on their lap. Thus, while not definitively a factor that prevented mothers from reducing their child's screen time, this theme suggests that mothers' personal screen use might subject their children to increased secondary or background screen time.

Facilitators that supported or empowered mothers to reduce or limit their child's screen use were also identified. These factors included sharing advice on how to limit screen time with other mothers (e.g., strategies, rules, techniques) and sharing examples of alternatives to screen use. A significant number of posts fell within these two subthemes which may indicate that social media platforms such as Reddit can positively support mothers in their quest to monitor and decrease their child's screen use. As described previously, mothers of young children may experience negative emotions of guilt or shame when sharing their personal parenting challenges with others, and Reddit offers an opportunity to share their experiences without fear of judgment [27, 28]. Many mothers explained that they were surrounded by messages from the media, family members or peers about the ills of child screen time and the recommended screen exposure for their child. Such widespread awareness and sharing of the risks associated with screen time could be perceived to help motivate mothers to take action to limit their child's screen use. However, most mothers were already aware of some of the health risks associated with screen use and felt they were trying their best to limit screen time as it was. Thus, receiving

continuous input from others often caused them to feel guilty or overwhelmed. The individuals or media sources expressing these messages regarding child screen time may have meant to help mothers. However, such input may be perceived by mothers as shaming those who allow screen time, which can adversely influence their mental health and subsequently impact the child [36].

Pre-pandemic, researchers examined the barriers and facilitators to reducing young children's screen time. Barriers identified in past research have included the ubiquitous nature of screens (e.g., at home, school, daycare), screen use within children's routine, the influence of other caregivers, the perceived addictive nature of screens, child enjoyment, lack of social network, weather, perceived health benefits, parent modelling or co-viewing, screen used as a babysitter, influence of other children, and parents' other responsibilities (e.g., working) [37, 38]. Compared to the results of the current study, some consistent barriers were noted, such as screens being embedded in the child's routine, high child screen use with other caregivers, using screens as a babysitter, and other commitments or obligations for the parent. Regarding routines, the current study results focused specifically on increased screen use practices adopted due to the pandemic. Moreover, using screens as a babysitter was reflected in two themes: mothers used screens to occupy their children while they attended to their other obligations or when they took a moment to rest or engage in self-care practices). Parent modelling or co-viewing (i.e., the child observing parent screen use or engaging in screens with the parent), a barrier identified in pre-pandemic research, is similar to theme five in the current study. In this theme, mothers described their children being exposed to background or secondary screen time. While mothers did not mention mutually enjoying screens with their child (i.e., co-viewing), many did explain that their child was often in the room while they were using a screen, such as watching television. During these instances, the mother may be modelling screen use for the child, and although the child

may not be attentively watching the screen, they may be glancing periodically, as was noted by some mothers.

Less surprising is that mothers' mental and physical health emerged as a new factor that limited their ability to reduce their child's screen time during the pandemic. Understandably, many posts regarding mothers' physical health influencing children's screen time were related to having COVID-19. Moreover, a study examining maternal mental health during the pandemic found that most mothers of young children experienced a decline in at least one aspect of their mental health [39]. Therefore, it is comprehensible that maternal mental health emerged as a new barrier to limiting young children's screen time. When mothers experienced anxiety, depression, exhaustion, or burnout, they often relied on their child's use of digital devices while they took a break to relax or rest, rather than having to entertain their children themselves. For example, mothers expressed allowing their children to watch TV while they attended a virtual psychotherapy session or engaged in their favourite exercise routine for self-care. This finding demonstrates that mothers' mental health may influence children's health behaviours, specifically screen time. Such information may inform future efforts that seek to reduce young children's screen time, including interventions that focus on promoting maternal mental health, particularly during precarious events such as pandemic that can profoundly affect psychological well-being [40].

Lastly, mothers' use of screens to soothe or calm their children or to encourage necessary behaviours (e.g., hygiene practices, eating) emerged as a new barrier not identified in pre-pandemic qualitative research. Mothers expressed that they offered their child more screen time if the child was acting fussy (i.e., whining, challenging to soothe), throwing a tantrum (i.e., extreme anger, upset, crying) or incentivize the child to eat or cooperate during hygienic

practices (i.e., nail trimming or bathing). This finding is intriguing, as Raghunathan and colleagues [41] found that during the COVID-19 pandemic, children demonstrated a significant decline in self-regulation ability. Lower self-regulation was indicated by children showing poorer concentration, task engagement and persistence, and greater impulsivity than they did before the pandemic [41]. A similar finding was noted by Katzman [42], who attributed the notable decline in emotional and self-regulation abilities in young children during the pandemic to their lack of social interaction and increased screen use. Lower capacity for children to monitor their behaviour, de-escalate heightened emotions, or cope with stress [43], which are characteristics of child self-regulation, may have resulted in mothers relying more on screens to calm their children. In addition, since high levels of screen use may decrease a child's self-regulation ability [5], it is necessary to consider how the use of screens to calm or soothe a child may not be helping a child regulate their behaviour or emotions. While screens may temporarily calm a child, as described by mothers, screens may be contributing to a decrease in children's ability to regulate their emotions or behaviour, potentially resulting in increased reliance on screens in the future.

Earlier research examining parents’ perceptions of their child’s screen use indicated that some believed screens offered health benefits to their children, such as allowing them to relax or regulating their sleep patterns [38]. In contrast, posts related to screen time in the current study indicated that most mothers were aware of some potential risks associated with child screen time. Despite being aware of these associations, several mothers wanted more information regarding the nuances of the risks involved, as seen in Theme 5 and Subtheme 2.5 in this study. For example, many were confused regarding which screens “counted” toward their child’s daily screen time and wanted to know the implications of background or secondary screen viewing and

specific communication apps like Facetime. This finding may indicate an existing knowledge gap. Public health messaging may have effectively spread awareness to mothers regarding child screen time risks and time recommendations; however, mothers desire a greater understanding of the types of screen exposure or content associated with the most significant health risks for their children.

Furthermore, widespread messaging regarding the risks of screen time and the influence of the media, peers, or other family members led several mothers to feel guilty when offering screen time to their children, as seen in Subtheme 2.7. With this understanding, it may be beneficial for future public health efforts to move away from absolute thinking or rigid time restrictions. Instead, translating practical strategies that consider our current context and reliance on screen use could help mothers promote their child's health and well-being within an increasingly virtual world. Recently, the CPS updated their screen time guidelines for children under five, moving away from rigid screen limits [1]. The CPS recommends avoiding screen time one hour before bed for children under five, prioritizing age-appropriate, educational, and interactive screen use, and prioritizing family media use over solitary screen use for children [1]. While such recommendations may allow mothers to make informed decisions regarding their children’s screen time, it is imperative that such information is properly disseminated and made accessible to all mothers.

Strengths and Limitations

A strength of this study is that anonymous discussion forums, like Reddit, allow people to engage in honest communication that may be difficult or sensitive to discuss in face-to-face settings [44, 45]. This study captured authentic emotions from mothers and offered unique insight into their perceptions regarding their young child’s screen use they may not have

otherwise shared, perhaps out of embarrassment or shame. However, the utilization of anonymous social media platforms for research does have limitations. Collecting demographic information of participants was not possible. Although population-level data have not been reported for individual subreddits, most users of the Reddit app are from the United States [46]. While it has been indicated that users are relatively representative of the American population, there are differences between the general public and Reddit users [46]. For example, Barthel [46] found that Reddit users were more educated than the general population. Moreover, Hargittai [47] argues that those of higher socioeconomic status are more likely to be on social media; and therefore, their views may be oversampled in big-data research, such as that collected on the Reddit platform. The perspectives captured in the current study may be skewed to represent unique challenges faced by mothers who are more educated and are from a higher socioeconomic background than the general population. In addition, the platform's anonymous nature can affect the content's validity. It is also possible for Reddit users to provide false or dishonest information [48].

Conclusion

The results indicate that young children’s screen time may have been influenced by mother’s mental and physical health experiences during the pandemic. In addition, a large portion of mothers relied on screens to soothe or calm their child or to incentivise them to engage in bedtime routines (e.g., bathing, teeth brushing) or other necessary behaviours. Many desire further information regarding which types of screen devices or content "count" towards their child's daily screen time or which pose the most significant health risk to their child. As technology becomes increasingly involved in children's everyday lives, interventions to reduce screen time should be tailored to address the needs voiced by caregivers who spend the most

time with their children. Thus, future efforts to reduce young children's screen time may aim to improve mothers' mental health and well-being, so they can better take action to reduce their child's screen time. Finally, this study demonstrates how social media platforms like Reddit may be utilized as a valuable source of public health information, despite being a novel or less studied tool for such knowledge, as researchers can learn from the first-hand experience of the population they wish to support or understand.

References

1. Canadian Paediatric Society. Screen time and young children: Promoting health and development in a digital world [Internet]. Ottawa, Ontario: Canadian Paediatric Society; 2017 Oct 9 [cited 2022 Nov]. https://cps.ca/en/documents/position/screen-time-and-young-children

2. Chonchaiya W, Pruksananonda C. Television viewing associates with delayed language development. Acta Paediatrica. 2008 Jul;97(7):977-82. https://doi.org/10.1111/j.1651-2227.2008.00831.x

3. Downing KL, Hnatiuk J, Hesketh KD. Prevalence of sedentary behavior in children under 2 years: a systematic review. Preventive medicine. 2015 Sep 1;78:105-14. https://doi.org/10.1016/j.ypmed.2015.07.019

4. Lin LY, Cherng RJ, Chen YJ, Chen YJ, Yang HM. Effects of television exposure on developmental skills among young children. Infant behavior and development. 2015 Feb 1;38:20-6. https://doi.org/10.1016/j.infbeh.2014.12.005

5. Radesky JS, Silverstein M, Zuckerman B, Christakis DA. Infant self-regulation and early childhood media exposure. Pediatrics. 2014 May;133(5):e1172-8. https://doi.org/10.1542/peds.2013-2367

6. Public Health Agency of Canada. Coronavirus disease (COVID-19): Canada's Response. Canada: Public Health Agency of Canada; 2020 [updated 2022 Oct 1]. Available from: https://www.canada.ca/en/public-health/services/diseases/2019-novel-coronavirus-infection/canadas-reponse.html

7. Toombs E, Mushquash CJ, Mah L, Short K, Young NL, Cheng C, Zhu L, Strudwick G, Birken C, Hopkins J. Increased screen time for children and youth during the COVID-19 pandemic. Sci. Briefs Ont. COVID-19 Sci. Advis. Table. 2022 Apr 12;3:59. https://doi.org/10.47326/ocsat.2022.03.59.1.0

8. Tamana SK, Ezeugwu V, Chikuma J, Lefebvre DL, Azad MB, Moraes TJ, Subbarao P, Becker AB, Turvey SE, Sears MR, Dick BD. Screen-time is associated with inattention problems in preschoolers: Results from the CHILD birth cohort study. PloS one. 2019 Apr 17;14(4):e0213995. https://doi.org/10.1371/journal.pone.0213995

9. Trott M, Driscoll R, Irlado E, Pardhan S. Changes and correlates of screen time in adults and children during the COVID-19 pandemic: A systematic review and meta-analysis. EClinicalMedicine. 2022 Jun 1;48:101452. https://doi.org/10.1016/j.eclinm.2022.101452

10. Lauricella, A. R., Wartella, E., & Rideout, V. J. (2015). Young children's screen time: The complex role of parent and child factors. *Journal of Applied Developmental Psychology*, *36*, 11-17. https://doi.org/10.1016/j.appdev.2014.12.001

11. Tips for setting screen time limits as a parent [Internet]. Mayo Clinic. 2022 [cited 2022 Nov]. Available from: https://www.mayoclinic.org/healthy-lifestyle/childrens-health/in-depth/screen-time/art-20047952

12. Jarvis JW, Harrington DW, Manson H. Exploring parent-reported barriers to supporting their child's health behaviors: A cross-sectional study. international journal of behavioral nutrition and physical activity. 2017 Dec;14:1-0. https://doi.org/10.1186/s12966-017-0508-9

13. Riazi NA, Wunderlich K, Gierc M, Brussoni M, Moore SA, Tremblay MS, Faulkner G. "You can't go to the park, you can't go here, you can't go there": Exploring parental experiences of COVID-19 and its impact on their children's movement behaviours. Children. 2021 Mar 12;8(3):219. https://doi.org/10.3390/children8030219

14. Drouin M, McDaniel BT, Pater J, Toscos T. How parents and their children used social media and technology at the beginning of the COVID-19 pandemic and associations with anxiety. Cyberpsychology, Behavior, and Social Networking. 2020 Nov 1;23(11):727-36. https://doi.org/10.1089/cyber.2020.0284

15. Tsao SF, Chen H, Tisseverasinghe T, Yang Y, Li L, Butt ZA. What social media told us in the time of COVID-19: a scoping review. Lancet Digit Health. 2021 Mar; 3 (3): e175–e194. https://doi.org/10.1016/S2589-7500(20)30315-0

16. Kinser P, Jallo N, Moyer S, Weinstock M, Barrett D, Mughal N, Stevens L, Rider A. "It's always hard being a mom, but the pandemic has made everything harder": A qualitative exploration of the experiences of perinatal women during the COVID-19 pandemic. Midwifery. 2022 Jun 1;109:103313. https://doi.org/10.1016/j.midw.2022.103313

17. Amaya A, Bach R, Keusch F, Kreuter F. New data sources in social science research: Things to know before working with Reddit data. Social science computer review. 2021 Oct;39(5):943-60. Available from: https://doi.org/10.1177/0894439319893305

18. Lee JY, Chang OD, Ammari T. Using social media Reddit data to examine foster families' concerns and needs during COVID-19. Child Abuse & Neglect. 2021 Nov 1;121:105262. https://doi.org/10.1016/j.chiabu.2021.105262

19. Sultana, A., Tasnim, S., Hossain, M. M., Bhattacharya, S., & Purohit, N. (2021). Digital screen time during the COVID-19 pandemic: a public health concern. *F1000Research*, *10*(81), 81. https://doi.org/10.12688/f1000research.50880.1

20. Wiederhold BK. Children's screen time during the COVID-19 pandemic: boundaries and etiquette. Cyberpsychology, Behavior, and Social Networking. 2020 Jun 1;23(6):359-60. https://doi.org/10.1089/cyber.2020.29185.bkw

21. Eyler AA, Schmidt L, Beck A, Gilbert A, Kepper M, Mazzucca S. Children's physical activity and screen time during COVID-19 pandemic: a qualitative exploration of parent perceptions. Health behavior and policy review. 2021 May 1;8(3):236-46. https://doi.org/10.14485/HBPR.8.3.5

22. Dunatchik A, Gerson K, Glass J, Jacobs JA, Stritzel H. Gender, parenting, and the rise of remote work during the pandemic: Implications for domestic inequality in the United States. Gender & Society. 2021 Apr;35(2):194-205. https://doi.org/10.1177/08912432211001301

23. Phelan KJ, Morrongiello BA, Khoury JC, Xu Y, Liddy S, Lanphear B. Maternal supervision of children during their first 3 years of life: the influence of maternal depression and child gender. Journal of pediatric psychology. 2014 Apr 1;39(3):349-57. https://doi.org/10.1093/jpepsy/jst090

24. Queiroz MM. A framework based on Twitter and big data analytics to enhance sustainability performance. Environmental Quality Management. 2018 Sep;28(1):95-100. https://doi.org/10.1002/tqem.21576

25. Griffith FJ, Redondo RA, Diggins E. Size, food, and sensuality: Predictors of network engagement in weight-inclusive Instagram communities. The Journal of Social Media in Society. 2022 Dec 30;11(2):278-95. http://orcid.org/0000-0001-9238-0212

26. Lin AE, Young JA, Guarino JE. Mother-Daughter sexual abuse: An exploratory study of the experiences of survivors of MDSA using Reddit. Children and youth services review. 2022 Jul 1;138:106497. https://doi.org/10.1016/j.childyouth.2022.106497

27. Constantinou G, Varela S, Buckby B. Reviewing the experiences of maternal guilt–the "Motherhood Myth" influence. Health Care for Women International. 2021 Jul 1;42(4-6):852-76. https://doi.org/10.1080/07399332.2020.1835917

28. Mitchell J. *Facing Motherhood Stressors: A Content Analysis of Advice-Giving Comments using the Subreddit r/breakingmom* (Doctoral dissertation, University of Nebraska at Omaha). https://www.proquest.com/docview/2682204988?pq-origsite=gscholar&fromopenview=true

29. Bengtsson M. How to plan and perform a qualitative study using content analysis. NursingPlus open. 2016 Jan 1;2:8-14. https://doi.org/10.1016/j.npls.2016.01.001

30. King M. Ontario school boards prep for back-to-school with additional supports for in-school learning [Internet]. Global News. Global News; 2022 [cited 2022 Nov]. Available from: https://globalnews.ca/news/9023584/ontario-school-boards-back-to-school-additional-supports/

31. Wu J, Tang B, Bragazzi NL, Nah K, McCarthy Z. Quantifying the role of social distancing, personal protection and case detection in mitigating COVID-19 outbreak in Ontario, Canada. Journal of mathematics in industry. 2020 Dec;10:1-2. https://doi.org/10.1186/s13362-020-00083-3

32. Gallagher-Mackay K, Srivastava P, Underwood K, Dhuey E, McCready L, Born K, Maltsev A, Perkhun A, Steiner R, Barrett K, Sander B. COVID-19 and education disruption in Ontario: Emerging evidence on impacts. https://scholars.wlu.ca/laso_faculty/1

33. Braun V, Clarke V. Using thematic analysis in psychology. Qualitative research in psychology. 2006 Jan 1;3(2):77-101. https://doi.org/10.1191/1478088706qp063oa

34. Lee HC, Arora V, Brown T, Lyndon A. Thematic analysis of barriers and facilitators to implementation of neonatal resuscitation guideline changes. Journal of Perinatology. 2017 Mar;37(3):249-53. https://doi.org/10.1038/jp.2016.217

35. Wong CW, Tsai A, Jonas JB, Ohno-Matsui K, Chen J, Ang M, Ting DS. Digital screen time during the COVID-19 pandemic: risk for a further myopia boom? American journal of ophthalmology. 2021 Mar 1;223:333-7. https://doi.org/10.1016/j.ajo.2020.07.034

36. Dawenan FN, Shanti P. The Correlation Between Husbands' Social Support and Anxiety in Mothers Who Have Experienced Mom-Shaming in Malang City. KnE Social Sciences. 2022 Jan 28:109-24. https://doi.org/10.18502/kss.v7i1.10205

37. Hesketh KD, Hinkley T, Campbell KJ. Children′ s physical activity and screen time: qualitative comparison of views of parents of infants and preschool children. International Journal of Behavioral Nutrition and Physical Activity. 2012 Dec;9(1):1-4. https://doi.org/10.1186/1479-5868-9-152

38. Minges KE, Owen N, Salmon J, Chao A, Dunstan DW, Whittemore R. Reducing youth screen time: qualitative metasynthesis of findings on barriers and facilitators. Health Psychology. 2015 Apr;34(4):381. https://doi.org/10.1037/hea0000172

39. Saleem S, Burns S, Falenchuk O, Varmuza P, Perlman M. Heterogeneity in maternal and child mental health responses to the COVID-19 pandemic. Early Childhood Research Quarterly. 2022 Apr 1;59:203-14. https://doi.org/10.1016/j.ecresq.2021.12.004

40. Holmes EA, O'Connor RC, Perry VH, Tracey I, Wessely S, Arseneault L, Ballard C, Christensen H, Silver RC, Everall I, Ford T. Multidisciplinary research priorities for the

COVID-19 pandemic: a call for action for mental health science. The Lancet Psychiatry. 2020 Jun 1;7(6):547-60. https://doi.org/10.1016/S2215-0366(20)30168-1

41. Raghunathan RS, Musci RJ, Voegtline KM, Thomas TC, Johnson SB. Children's Attention and Self-Regulatory Behavior before and during the COVID-19 Pandemic. Journal of developmental and behavioral pediatrics: JDBP. 2022 May 5;43(4):e263. https://doi.org/10.1097/DBP.0000000000001027

42. Katzman R. *Parenting Practices, Technology Use, and Preschoolers' Self-Regulation During COVID-19: A Thematic Analysis* (Doctoral dissertation, University of Windsor (Canada)). Available from: https://www.proquest.com/docview/2728434116?fromopenview=true&pq-origsite=gscholar

43. Gus L, Rose J, Gilbert L. Emotion coaching: A universal strategy for supporting and promoting sustainable emotional and behavioural well-being. Educational & Child Psychology. 2015 Mar;32(1):31-41. Available from: https://www.academia.edu/81148530/Emotion_Coaching_A_universal_strategy_for_supporting_and_promoting_sustainable_emotional_and_behavioural_well_being?sm=b

44. Sillence E. Giving and receiving peer advice in an online breast cancer support group. Cyberpsychology, Behavior, and Social Networking. 2013 Jun 1;16(6):480-5. https://doi.org/10.1089/cyber.2013.1512

45. White MH, Dorman SM. Online support for caregivers. Analysis of an Internet Alzheimer mailgroup. Computers in nursing. 2000 Jul 1;18(4):168-76.Available from: https://oce-ovid-com.proxy1.lib.uwo.ca/article/00002771-200007000-00013?sequence=0&clickthrough=y

46. Barthel, M., Stocking, G., Holcomb, J., & Mitchell, A. Seven-in-ten Reddit users get news on the site [Internet]. United States of America: Pew Research Center's Journalism Project; 2016 Feb 25 [cited 2022 Nov]. Available from: https://policycommons.net/artifacts/618607/seven-in-ten-reddit-users-get-news-on-the-site/1599577/

47. Hargittai E. Potential biases in big data: Omitted voices on social media. Social Science Computer Review. 2020 Feb;38(1):10-24. https://doi.org/10.1177/0894439318788322

48. Callaghan S, Drysdale MT, Lee J. Wellness, blaming and coping during a pandemic: an analysis of perceptions on reddit. Mental Health and Social Inclusion. 2021 Jul 22;25(3):267-78. https://doi.org/10.1108/MHSI-05-2021-0021

Figure 1

Mothers' Perceived Barriers to Reducing Young Children's Screen Time During the COVID-19 Pandemic.

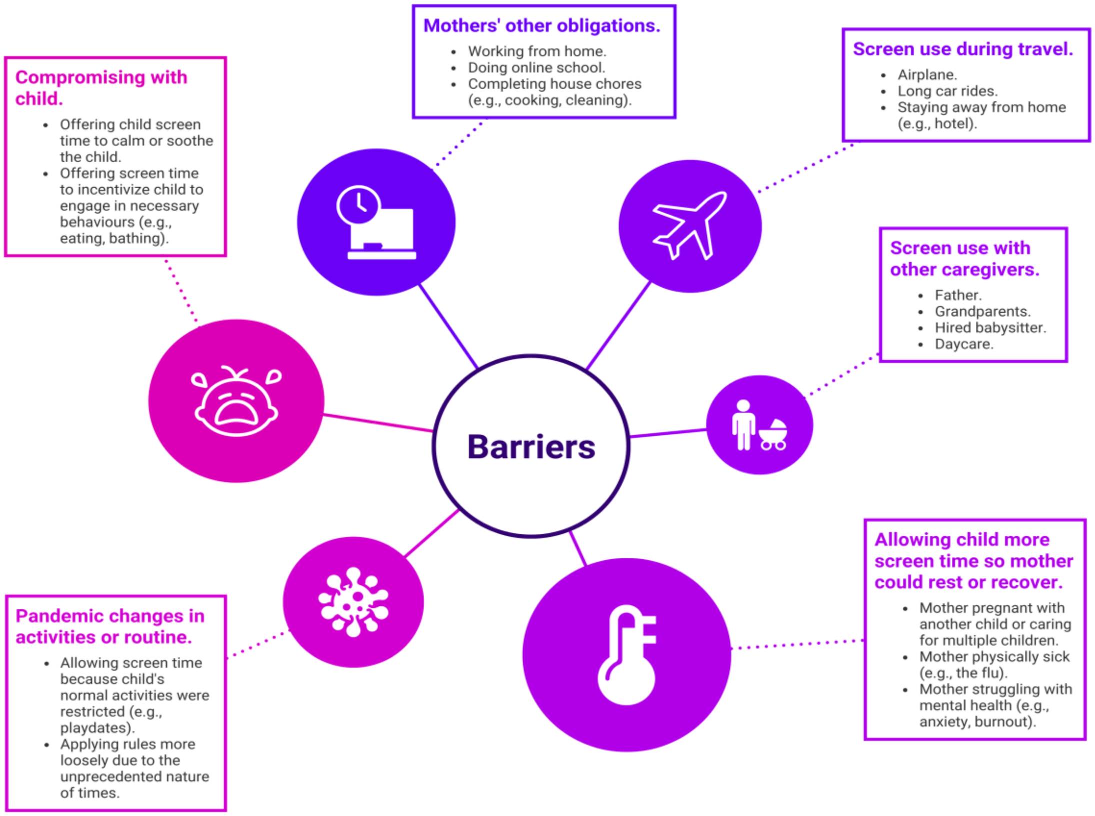

Note: The size of the circles represent the number of posts (i.e., magnitude) discussing each barrier.
