# Supplementary material for: Mothers’ perspectives of the barriers and facilitators to reducing young children’s screen time during COVID-19: A reddit content analysis
Source: PLoS One. 2024 Mar 27;19(3):e0301089. doi: 10.1371/journal.pone.0301089 (PMC10971781; doi:10.1371/journal.pone.0301089)
Supplement: Attachment — Submitted filename: Response to reviewers - Feb 19.docx [file pone.0301089.s002.docx]

**Response to Reviewers – Manuscript ID [PONE-D-23-18207]**

February 19, 2024

Dear Dr. Kalembo,

Thank you for reviewing our manuscript. We appreciate the time the reviewers have given us in terms of their constructive feedback on our manuscript. We have applied the feedback and comments and would like to submit a revised version of the manuscript for further consideration in the journal. Changes in the original version of the manuscript have been tracked, and we have provided point-by point responses below to explain how we have addressed comments from the editor.

We look forward to your review of our manuscript and to the outcome of your evaluation. Thank you.

Sincerely,

Dr. Leigh Vanderloo

Adjunct Professor

School of Occupational Therapy

Western University

Email: [lvande32@uwo.ca](mailto:lvande32@uwo.ca)

**Journal Requirements:**

1. We note your current data availability statement reads as follows:

   "The data described and analyzed in this article are openly available on the Reddit platform at <https://www.reddit.com/r/Mommit/> and [https://www.reddit.com/r/beyondthebump/."](https://www.reddit.com/r/beyondthebump/.%22)

   However, Reddit is not an acceptable data sharing platform.

   To ensure you submission adheres to the PLOS ONE Data Availability policy (<https://journals.plos.org/plosone/s/data-availability>), please either upload the minimal anonymized data set required to replicate your study’s findings as Supporting Information, or to a stable public repository. Please also provide all relevant DOIs, URLs, and or/accession numbers required to access your data on the repository. For a list of acceptable repositories, please see <https://journals.plos.org/plosone/s/recommended-repositories>.

- **Action taken:** The data described and analyzed in this article are openly available on from Western University's Institutional Open-Access Repository (Borealis): <https://borealisdata.ca/dataset.xhtml?persistentId=doi:10.5683/SP3/RLC7FO>.

The above sentence has been included in the first paragraph of the “Methods” section of the paper (page 7).
